# CALLY index predicts survival and surgical outcomes in colorectal cancer

**DOI:** 10.3389/fnut.2025.1723789

**Published:** 2025-12-17

**Authors:** Yunmeng Zong, Yulong Wang, Ying Chen, Xiao Feng, Bin Jiang, Yun Li

**Affiliations:** Department of Gastrointestinal Surgery, Xinghua People's Hospital Affiliated to Yangzhou University, Xinghua, China

**Keywords:** colorectal cancer, CALLY index, prognostic biomarker, survival analysis, systemic inflammation

## Abstract

**Background:**

The prognostic evaluation of colorectal cancer (CRC) traditionally relies on TNM staging, which fails to incorporate host-related factors such as systemic inflammation, nutrition, and immunity. The C-reactive protein–albumin–lymphocyte (CALLY) index has recently been proposed as a novel biomarker integrating these domains.

**Methods:**

We retrospectively analyzed 957 patients with CRC undergoing curative resection (2010–2020). The CALLY index was calculated from preoperative laboratory data. Patients were stratified into high- and low-CALLY groups using ROC-derived cutoffs. Associations with postoperative complications, overall survival (OS), and disease-free survival (DFS) were assessed and compared with other indices (mGPS, PNI, NLR, PLR, SII, CAR).

**Results:**

Low CALLY was significantly associated with higher complication rates (23.0% vs. 14.9%, *p* = 0.002), inferior OS and DFS (both log-rank *p* < 0.001), and remained an independent predictor in multivariable Cox and logistic models. Compared with other indices, CALLY demonstrated stronger discriminatory ability, achieving the highest AUC for 5-year OS, and its C-index value also outperformed other indices, further confirming the predictive efficacy of CALLY.

**Conclusion:**

The preoperative CALLY index is a simple, cost-effective, and reliable prognostic biomarker for CRC, predicting both surgical outcomes and long-term survival. Incorporation of CALLY into risk stratification may complement TNM staging, optimize perioperative management, and inform individualized treatment strategies. Further validation in multicenter, prospective cohorts is required to confirm the generalizability of these findings.

## 1 Introduction

Colorectal cancer (CRC) is among the most common and lethal malignancies worldwide, ranking third in incidence and second in cancer-related mortality (1). With the ongoing shift in lifestyle patterns, its burden continues to rise in both developed and developing regions (2). Despite remarkable advances in surgical techniques, perioperative management, and systemic therapies, the overall prognosis of CRC remains unsatisfactory. A substantial proportion of patients still experience postoperative complications, recurrence, and poor long-term survival (3). This highlights the limitations of current prognostic tools and underscores the urgent need for more accurate and comprehensive risk stratification systems.

At present, prognostic evaluation in clinical practice relies mainly on the tumor–node-metastasis (TNM) staging system. While TNM staging serves as the cornerstone of oncological decision-making by reflecting tumor burden and anatomical extent, it fails to adequately incorporate host-related biological factors. Increasing evidence suggests that host conditions-including nutritional status, systemic inflammation, and immune competence-play critical roles in surgical tolerance, postoperative morbidity, recurrence risk, and overall survival (4). However, these factors are insufficiently integrated into conventional prognostic models, partially explaining the marked heterogeneity in outcomes among patients within the same TNM stage.

Against this background, composite indices that integrate inflammatory, nutritional, and immune parameters have attracted growing attention. Among them, the C-reactive protein-albumin-lymphocyte (CALLY) index has recently emerged as a promising prognostic biomarker (5). This index combines three routinely available laboratory markers: C-reactive protein (CRP), a surrogate of systemic inflammation and tumor-promoting milieu; serum albumin, an indicator of nutritional and metabolic reserve; and lymphocyte count, a reflection of immune competence and antitumor capacity. By consolidating these parameters into a single score, the CALLY index provides a more comprehensive representation of patient physiological status than single-factor measures, bridging the gap between tumor-centered staging and host-related prognostic factors.

Evidence from various malignancies-including gastric, hepatocellular, and esophageal cancers-has demonstrated that a low CALLY index is associated with adverse prognosis (6–9). Patients with low scores often exhibit poorer overall and disease-free survival, higher rates of postoperative complications, delayed recovery, and reduced tolerance to adjuvant therapies. These findings suggest that the CALLY index may serve as a simple yet powerful risk stratification tool applicable across multiple clinical settings (10). Compared with other inflammation- and nutrition-based scores, such as the neutrophil-to-lymphocyte ratio (NLR), platelet-to-lymphocyte ratio (PLR), and prognostic nutritional index (PNI), the CALLY index has shown potentially superior or complementary predictive performance in studies of gastrointestinal cancers (8, 11). Notably, as it is derived entirely from routine blood tests, the CALLY index is cost-effective, noninvasive, and easily implemented in daily practice without imposing additional burden on patients.

However, in the field of CRC, evidence supporting the prognostic value of the CALLY index remains limited and fragmented. Few studies have systematically evaluated its association with surgical outcomes, recurrence, and long-term survival, or directly compared its performance against existing prognostic tools (12–14). It remains unclear whether incorporating the CALLY index into preoperative assessment could meaningfully alter clinical decision-making-for example, by identifying high-risk patients who may benefit from more intensive perioperative management, nutritional optimization, or tailored adjuvant therapies.

Therefore, further systematic validation is essential to establish the clinical utility of the CALLY index in CRC. Comprehensive assessment of its independent prognostic significance and comparative performance with existing models will not only refine individualized treatment strategies but also facilitate more efficient allocation of medical resources, ultimately improving the overall prognosis of patients with CRC.

## 2 Methods

### 2.1 Study design and patients

This retrospective cohort study included patients with pathologically confirmed colorectal adenocarcinoma who underwent curative resection at Xinghua People’s Hospital from January 2010 to December 2020. Inclusion criteria were R0 resection and availability of complete preoperative clinical and laboratory data. Exclusion criteria were: distant metastasis at diagnosis; active inflammatory, autoimmune, hematologic, or infectious diseases that could affect biomarkers; perioperative death within 30 days; or incomplete medical records.

The study was approved by the Ethics Committee of Xinghua People’s Hospital Affiliated to Yangzhou University. Given that this is a retrospective data analysis, with all data derived from previously recorded medical charts, the study was exempt from obtaining informed consent as per the regulations of the Ethics Committee.

### 2.2 Data collection and variables

Clinical, pathological, and laboratory data were extracted from electronic medical records. Preoperative laboratory tests obtained within 7 days before surgery included serum CRP, albumin, and peripheral absolute lymphocyte count. The CALLY index was calculated as: CALLY = (Albumin [g/dL] × Lymphocyte count [/μL])/(CRP [mg/dL] × 10^4^).

In addition, several inflammation- and nutrition-based biomarkers were derived from the same preoperative laboratory data to comprehensively evaluate systemic immune and nutritional status. These included the mGPS, PNI, PLR, SII, and CAR. The mGPS was determined according to serum CRP and albumin levels, while the PNI integrated albumin concentration with lymphocyte count. Ratios such as NLR, PLR, and SII reflected the balance between inflammatory and immune cell components, whereas CAR represented the relationship between CRP and albumin.

Other variables included age, sex, body mass index (BMI), tumor site and size, histological grade, vascular and perineural invasion, TNM stage, and perioperative information. If original units differed, values were converted to the units above prior to calculation. Lymphocyte count was converted from /μL to ×10^9/L using the conversion factor: 1/μL = 0.001 × 10^9/L. All data were adjusted accordingly.

The assessment of postoperative complications and survival outcomes was not performed by clinicians blinded to the CALLY index groups. Although blinding was not employed in this study, we minimized potential ascertainment bias by relying on routine clinical data (such as medical records, laboratory results, etc.).

### 2.3 Outcomes

Primary outcomes were overall survival (OS; time from surgery to death from any cause) and disease-free survival (DFS; time from surgery to first recurrence or death), confirmed via clinic visits and medical records. Postoperative complications within 30 days were recorded according to the Clavien–Dindo classification.

### 2.4 Statistical analysis

The optimal CALLY cutoff was determined using receiver operating characteristic (ROC) analysis with Youden’s index, based on 5-year OS data. The optimal cutoff was defined as the point that maximized the sum of sensitivity and specificity, used to predict long-term survival outcomes in CRC patients. To assess the stability and reliability of the cutoff value, Bootstrapping analysis was performed with 1,000 resamples. Based on this cutoff, patients were categorized into high-CALLY and low-CALLY groups. The prognostic value of the CALLY index was assessed by comparing OS and DFS between the two groups using Kaplan–Meier survival curves and log-rank tests. Continuous variables were summarized as mean ± standard deviation or median (interquartile range) and compared using the t test or Mann–Whitney U test, as appropriate. Categorical variables were expressed as counts and compared using the chi-square test or Fisher’s exact test. The proportional hazards assumption for the Cox model was validated using the Schoenfeld residuals test. The results of the test showed no significant correlation between the residuals and time (*p* > 0.05), indicating that the proportional hazards assumption was satisfied. Therefore, the Cox regression model was considered appropriate for the analysis. Cox proportional hazards models were used to identify independent prognostic factors. Logistic regression was used to analyze risk factors for postoperative complications. Variables with statistical significance in univariate analysis (*p* < 0.05) were included in the multivariate analysis. No missing data were identified, and all continuous and categorical variables were complete in the study.

The prognostic performance of CALLY was compared with that of NLR, PLR, PNI, modified Glasgow Prognostic Score (mGPS), systemic immune–inflammation index (SII), and C-reactive protein-to-albumin ratio (CAR) using the concordance index (C-index), time-dependent area under the curve (AUC), and calibration.

## 3 Results

### 3.1 Patient characteristics

**Figure 1:**
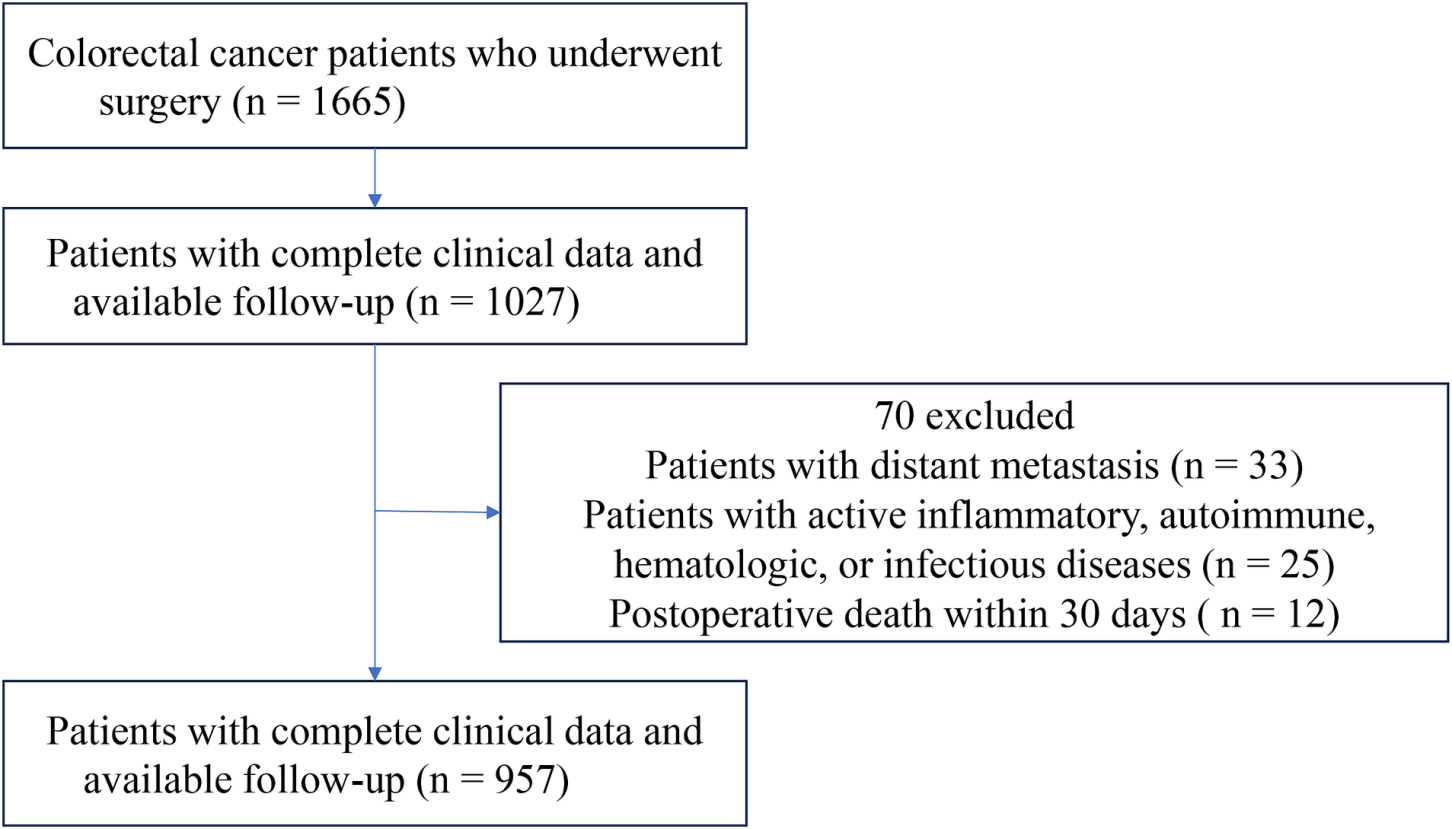
Patient selection flowchart.

This study included 957 patients with CRC (Figure 1), of whom 265 (27.7%) were classified into the low CALLY group (≤ 4.66) and 692 (72.3%) into the high CALLY group (> 4.66) according to the optimal ROC-derived cutoff. This cutoff was validated by bootstrapping analysis with 1,000 resamples, confirming its stability. Baseline clinicopathological features are presented in Table 1. Compared with the high CALLY group, patients in the low CALLY group were older, had lower albumin and lymphocyte levels, and higher CRP and neutrophil counts (all *p* < 0.001). Differences were also noted in histologic type, neoadjuvant chemotherapy, and adjuvant chemotherapy.

**Table 1:** Clinical and pathological characteristics between low and high CALLY index groups.

Variables	CALLY ≤ 4.66 (*n* = 265)	CALLY > 4.66 (*n* = 692)	*p*-value
Age, years; median (IQR)	72 (56–80)	66 (52–79)	<0.001
Sex [*n* (%)]			0.284
Male	137 (51.7)	379 (54.8)	
Female	128 (48.3)	313 (45.2)	
BMI, kg/m^2^	22.1 ± 2.0	22.6 ± 1.8	0.121
Albumin, g/dL; median (IQR)	3.5 (3.2–3.8)	4.1 (3.8–4.3)	<0.001
Lymphocyte (×10^9^/L); median (IQR)	1.2 (0.9–1.5)	1.7 (1.4–2.0)	<0.001
CRP, mg/L; median (IQR)	11.2 (5.0–26.8)	1.0 (0.5–2.3)	<0.001
Neutrocyte (×10^9^/L); median (IQR)	3.3 (2.5–4.3)	4.7 (3.4–6.5)	<0.001
Platelet (×10^9^/L); median (IQR)	198 (160–252)	230 (176–295)	<0.001
TNM stage			<0.001
I	40 (15.1)	173 (25.0)	
II	77 (29.1)	171 (24.7)	
III	135 (50.9)	248 (50.3)	
Operation method			0.518
Open	31 (11.7)	71 (10.3)	
Laparoscopy	234 (88.5)	621 (89.7)	
Histologic type			0.045
Differentiated	237 (89.4)	337 (93.0)	
Undifferentiated	28 (10.6)	27 (7.0)	
Neoadjuvant chemotherapy			0.014
Yes	40 (15.1)	66 (9.5)	
No	225 (84.9)	626 (90.5)	
Adjuvant chemotherapy			0.002
Yes	172 (64.9)	380 (54.9)	
No	93 (35.1)	312 (45.1)	

CALLY, C-reactive protein-albumin-lymphocyte; TNM, Tumor-Node-Metastasis classification. Lymphocyte count is presented in ×10^9/L, converted from the original/μL measurements (1/μL = 0.001 × 10^9/L).

### 3.2 Postoperative complications

As shown in Table 2, postoperative complications occurred more frequently in the low CALLY group than in the high CALLY group (23.0% vs. 14.9%, *p* = 0.002). The distribution of intestinal, infectious, and urinary events was similar between groups; however, severe complications (Clavien–Dindo grade III–V) were almost twice as common in the low CALLY group (6.0% vs. 2.9%, *p* = 0.006).

**Table 2:** Postoperative complications in low and high CALLY index groups.

Complications	CALLY≤4.66 (*n* = 265)	CALLY>4.66 (*n* = 692)	*p*-value
Total complications	61 (23.0)	103 (14.9)	0.002
Intestinal complications			
Ileus	8 (3.0)	15 (2.2)	
Delayed bowel function recovery	13 (4.9)	20 (2.9)	
Anastomotic leakage	5 (1.9)	7 (1.0)	
Anastomotic stenosis	1 (0.4)	2 (0.3)	
Anastomotic bleeding	3 (1.1)	6 (0.9)	
Infectious complications			
Intra-abdominal infection/abscess	6 (2.3)	10 (1.4)	
Pulmonary infection	14 (5.3)	23 (3.3)	
Wound infection	6 (2.3)	8 (1.2)	
Urinary complications			
Urinary tract infection/retention	3 (1.1)	6 (0.9)	
Other complications			
Lymphorrhagia	0 (0.0)	1 (0.1)	
Venous thromboembolism	1 (0.4)	2 (0.3)	
Cardiovascular events	1 (0.4)	3 (0.4)	
Severity (Clavien–Dindo classification)			
Minor (grade I-II)	45 (17.0)	83 (12.0)	0.048
Major (grade III-V)	16 (6.0)	20 (2.9)	0.006

CALLY, C-reactive protein-albumin-lymphocyte.

### 3.3 Prognostic significance of the CALLY index

**Figure 2:**
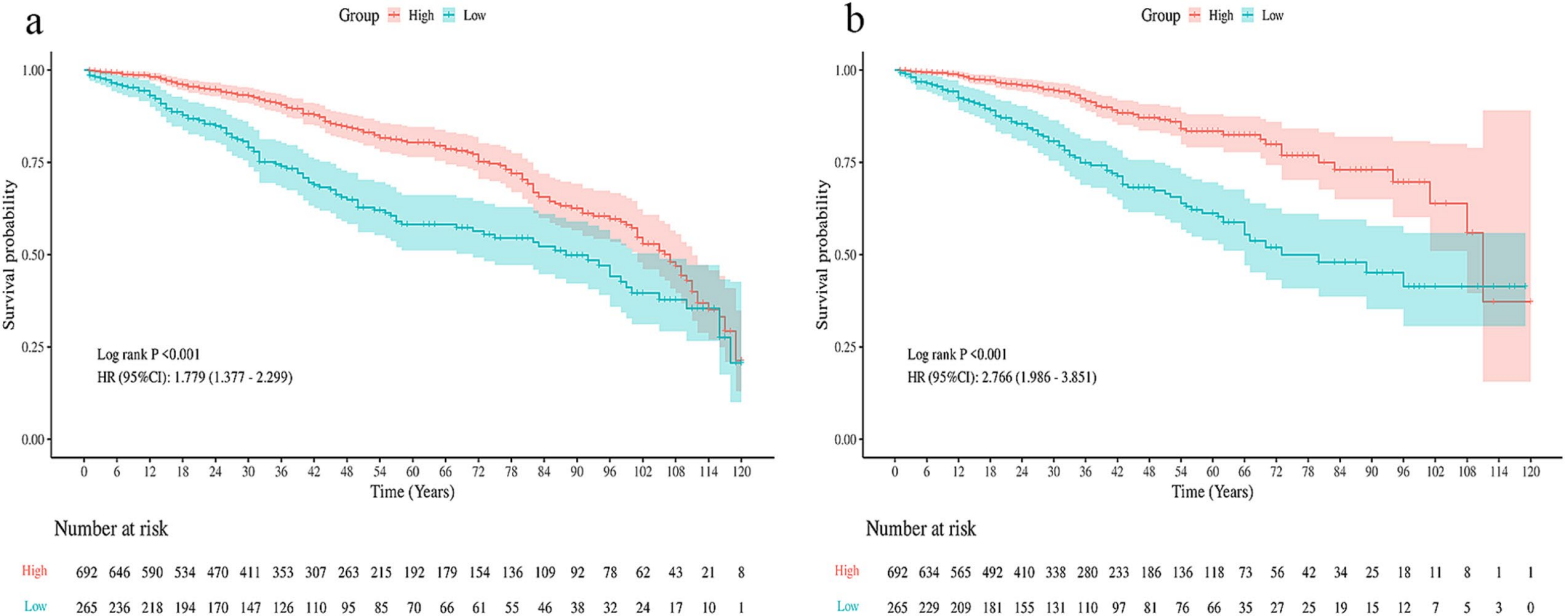
Kaplan–Meier survival curves stratified by the CALLY index. **(a)** Overall survival (OS) curves according to the CALLY index. **(b)** Disease-free survival (DFS) curves according to the CALLY index.

Kaplan–Meier curves demonstrated inferior OS and DFS in the low CALLY group compared with the high CALLY group (both log-rank *p* < 0.001) (Figures 2A,B). In Cox analysis, low CALLY was linked to unfavorable OS (HR 1.78, 95% CI 1.38–2.30, *p* < 0.001) and DFS (HR 1.85, 95% CI 1.44–2.38, *p* < 0.001). After adjusting for TNM stage and histology, low CALLY remained an independent predictor of poor OS (HR 1.49, 95% CI 1.15–1.94, *p* = 0.003) and DFS (HR 1.42, 95% CI 1.10–1.83, *p* = 0.005) (Tables 3, 4).

**Table 3:** Univariable and multivariable Cox regression analyses of prognostic factors for overall survival.

Variables	Univariable analysis			Multivariate analysis		
	HR	95% CI	*p*-value	HR	95% CI	*p*-value
Age	1.02	0.99–1.05	0.120			
Sex						
Male	1					
Female	0.95	0.77–1.18	0.642			
BMI	0.99	0.96–1.02	0.314			
TNM stage						
I	1			1		
II	1.58	1.10–2.27	0.013	1.44	1.00–2.07	0.048
III	3.05	2.19–4.25	<0.001	2.30	1.65–3.20	<0.001
Operation method						
Open	1					
Laparoscopy	0.92	0.74–1.14	0.453			
Histologic type						
Differentiated	1			1		
Undifferentiated	1.36	1.04–1.78	0.024	1.32	1.02–1.71	0.036
Neoadjuvant chemotherapy						
No	1					
Yes	1.08	0.82–1.44	0.582			
Adjuvant chemotherapy						
No	1					
Yes	0.92	0.72–1.18	0.518			
CALLY						
High	1			1		
Low	1.78	1.38–2.30	<0.001	1.49	1.15–1.94	0.003

CALLY, C-reactive protein-albumin-lymphocyte.

**Table 4:** Univariable and multivariable Cox regression analyses of prognostic factors for disease-free survival.

Variables	Univariable analysis			Multivariate analysis		
	HR	95% CI	*p*-value	HR	95% CI	*p*-value
Age	1.01	0.99–1.04	0.224			
Sex						
Male	1					
Female	0.97	0.78–1.21	0.768			
BMI	0.98	0.95–1.02	0.312			
TNM stage						
I	1			1		
II	1.68	1.20–2.35	0.003	1.47	1.05–2.06	0.025
III	3.28	2.41–4.47	<0.001	2.55	1.84–3.53	<0.001
Operation method						
Open	1					
Laparoscopy	0.95	0.75–1.21	0.681			
Histologic type						
Differentiated	1			1		
Undifferentiated	1.41	1.09–1.83	0.009	1.30	1.02–1.66	0.035
Neoadjuvant chemotherapy						
No	1					
Yes	1.10	0.82–1.48	0.529			
Adjuvant chemotherapy						
No	1					
Yes	0.87	0.68–1.11	0.259			
CALLY						
High	1			1		
Low	1.85	1.44–2.38	<0.001	1.42	1.10–1.83	0.005

CALLY, C-reactive protein-albumin-lymphocyte.

### 3.4 Determinants of postoperative complications

Multivariable logistic regression identified low CALLY as an independent risk factor for both overall complications (OR 1.80, 95% CI 1.32–2.45, *p* < 0.001) and severe complications (OR 1.85, 95% CI 1.30–2.65, *p* = 0.003) (Tables 5, 6). Advanced age and higher BMI also increased complication risk.

**Table 5:** Univariable and multivariable logistic regression analyses of risk factors for postoperative complications.

Variables	Univariable analysis			Multivariate analysis		
	OR	95% CI	*p*-value	OR	95% CI	*p*-value
Age	1.04	1.02–1.07	0.002	1.03	1.01–1.06	0.009
Sex						
Male	1					
Female	0.94	0.76–1.16	0.552			
BMI	1.09	1.03–1.15	0.004	1.07	1.01–1.13	0.012
TNM stage						
I	1			1		
II	1.52	1.08–2.14	0.017	1.40	1.01–1.96	0.043
III	2.25	1.60–3.16	<0.001	2.05	1.42–2.94	<0.001
Operation method						
Open	1			1		
Laparoscopy	0.70	0.55–0.90	0.008	0.78	0.60–1.01	0.061
Histologic type						
Differentiated	1					
Undifferentiated	1.12	0.84–1.50	0.464			
Neoadjuvant chemotherapy						
No	1					
Yes	1.12	0.85–1.47	0.412			
Adjuvant chemotherapy						
No	1					
Yes	0.95	0.72–1.28	0.767			
CALLY						
High	1			1		
Low	1.95	1.43–2.65	<0.001	1.80	1.32–2.45	<0.001

CALLY, C-reactive protein-albumin-lymphocyte.

**Table 6:** Univariable and multivariable logistic regression analyses of risk factors for severe postoperative complications.

Variables	Univariable analysis			Multivariate analysis		
	OR	95% CI	*p*-value	OR	95% CI	*p*-value
Age	1.05	1.02–1.08	0.001	1.04	1.01–1.07	0.012
Sex						
Male	1					
Female	0.90	0.65–1.25	0.528			
BMI	1.06	1.00–1.12	0.048	1.05	0.99–1.11	0.11
TNM stage						
I	1			1		
II	1.60	1.05–2.45	0.029	1.42	0.96–2.12	0.078
III	2.80	1.90–4.15	<0.001	2.35	1.58–3.50	<0.001
Operation method						
Open	1					
Laparoscopy	0.75	0.55–1.03	0.072			
Histologic type						
Differentiated	1					
Undifferentiated	1.20	0.82–1.76	0.352			
Neoadjuvant chemotherapy						
No	1					
Yes	1.18	0.82–1.70	0.367			
Adjuvant chemotherapy						
No	1					
Yes	0.92	0.66–1.30	0.639			
CALLY						
High	1					
Low	2.10	1.50–2.95	<0.001	1.85	1.30–2.65	0.003

CALLY, C-reactive protein-albumin-lymphocyte.

### 3.5 Comparison with alternative scores

We next compared CALLY with other inflammation- and nutrition-based indices, including mGPS, PNI, NLR, PLR, SII, and CAR. In univariate models, all markers were associated with OS and DFS. However, in adjusted analyses, only CALLY consistently retained prognostic value, while most other scores lost significance (Table 7). Similar findings were observed for postoperative outcomes, with CALLY but not other indices independently predicting overall and severe complications (Table 8).

**Table 7:** Comparison of univariate and multivariate analyses of OS and DFS for the CALLY index, mGPS, PNI, NLR, PLR, SII, and CAR.

Variables	Univariable analysis		Multivariable analysis		Univariable analysis		Multivariable analysis	
	OS HR	*P*	OS HR	*P*	DFS HR	*P*	DFS HR	*P*
CALLY								
High	1		1		1		1	
Low	1.95 (1.52–2.50)	<0.001	1.62 (1.26–2.08)	0.001	2.08 (1.63–2.66)	<0.001	1.55 (1.21–1.99)	<0.001
NLR								
Low	1		1		1		1	
High	1.32 (1.05–1.67)	0.016	1.19 (0.89–1.60)	0.243	1.35 (1.07–1.72)	0.011	1.18 (0.87–1.60)	0.259
PLR								
Low	1		1		1		1	
High	1.25 (1.01–1.57)	0.037	1.14 (0.83–1.56)	0.386	1.28 (1.03–1.61)	0.027	1.12 (0.81–1.55)	0.447
PNI								
Low	1		1		1		1	
High	1.36 (1.07–1.74)	0.012	1.20 (0.90–1.60)	0.192	1.40 (1.10–1.79)	0.006	1.23 (0.93–1.64)	0.137
mGPS								
0.1	1		1		1		1	
2	1.78 (1.27–2.50)	0.001	1.62 (1.02–2.57)	0.041	1.73 (1.23–2.44)	0.002	1.55 (1.00–2.42)	0.048
SII								
Low	1		1		1		1	
High	1.30 (1.02–1.67)	0.032	1.17 (0.86–1.59)	0.278	1.34 (1.06–1.72)	0.015	1.19 (0.88–1.61)	0.247
CAR								
Low	1		1		1		1	
High	1.45 (1.10–1.92)	0.009	1.23 (0.91–1.67)	0.162	1.47 (1.12–1.95)	0.005	1.25 (0.93–1.70)	0.129

CALLY, C-reactive protein-albumin-lymphocyte; NLR, neutrophil-to-lymphocyte ratio; PLR, platelet-to-lymphocyte ratio; PNI, prognostic nutritional index; mGPS, modified Glasgow prognostic score; SSI, systemic immune-inflammation index; CAR, C-reactive protein/albumin ratio.

**Table 8:** Comparison of univariate and multivariate analyses of complications and severe complications for the CALLY index, mGPS, PNI, NLR, and PLR.

Variables	Univariable analysis		Multivariable analysis		Univariable analysis		Multivariable analysis	
	Complications OR	*P*	Complications OR	*P*	Severe complications OR	*P*	Severe complications OR	*P*
CALLY								
High	1		1		1		1	
Low	1.78 (1.40–2.26)	<0.001	1.48 (1.15–1.90)	0.002	2.05 (1.52–2.76)	<0.001	1.65 (1.20–2.28)	0.002
NLR								
Low	1		1		1		1	
High	1.30 (1.02–1.66)	0.036	1.16 (0.88–1.52)	0.314	1.38 (1.07–1.78)	0.014	1.21 (0.90–1.62)	0.219
PLR								
Low	1		1		1		1	
High	1.32 (1.03–1.70)	0.028	1.15 (0.86–1.55)	0.333	1.40 (1.06–1.85)	0.019	1.18 (0.88–1.59)	0.243
PNI								
Low	1		1		1		1	
High	1.38 (1.08–1.76)	0.011	1.20 (0.92–1.57)	0.187	1.48 (1.13–1.95)	0.005	1.25 (0.94–1.67)	0.124
mGPS								
0.1	1		1		1		1	
2	1.65 (1.22–2.24)	0.002	1.40 (1.01–1.95)	0.045	1.87 (1.33–2.64)	<0.001	1.56 (1.05–2.30)	0.029
SII								
Low	1		1		1		1	
High	1.29 (1.01–1.65)	0.038	1.15 (0.85–1.56)	0.318	1.36 (1.05–1.77)	0.016	1.19 (0.88–1.61)	0.233
CAR								
Low	1		1		1		1	
High	1.43 (1.08–1.90)	0.013	1.22 (0.90–1.65)	0.178	1.48 (1.12–1.96)	0.007	1.26 (0.93–1.71)	0.131

CALLY, C-reactive protein-albumin-lymphocyte; NLR, neutrophil-to-lymphocyte ratio; PLR, platelet-to-lymphocyte ratio; PNI, prognostic nutritional index; mGPS, modified Glasgow prognostic score; SSI, systemic immune-inflammation index; CAR, C-reactive protein/albumin ratio.

### 3.6 Discriminative capacity

**Figure 3:**
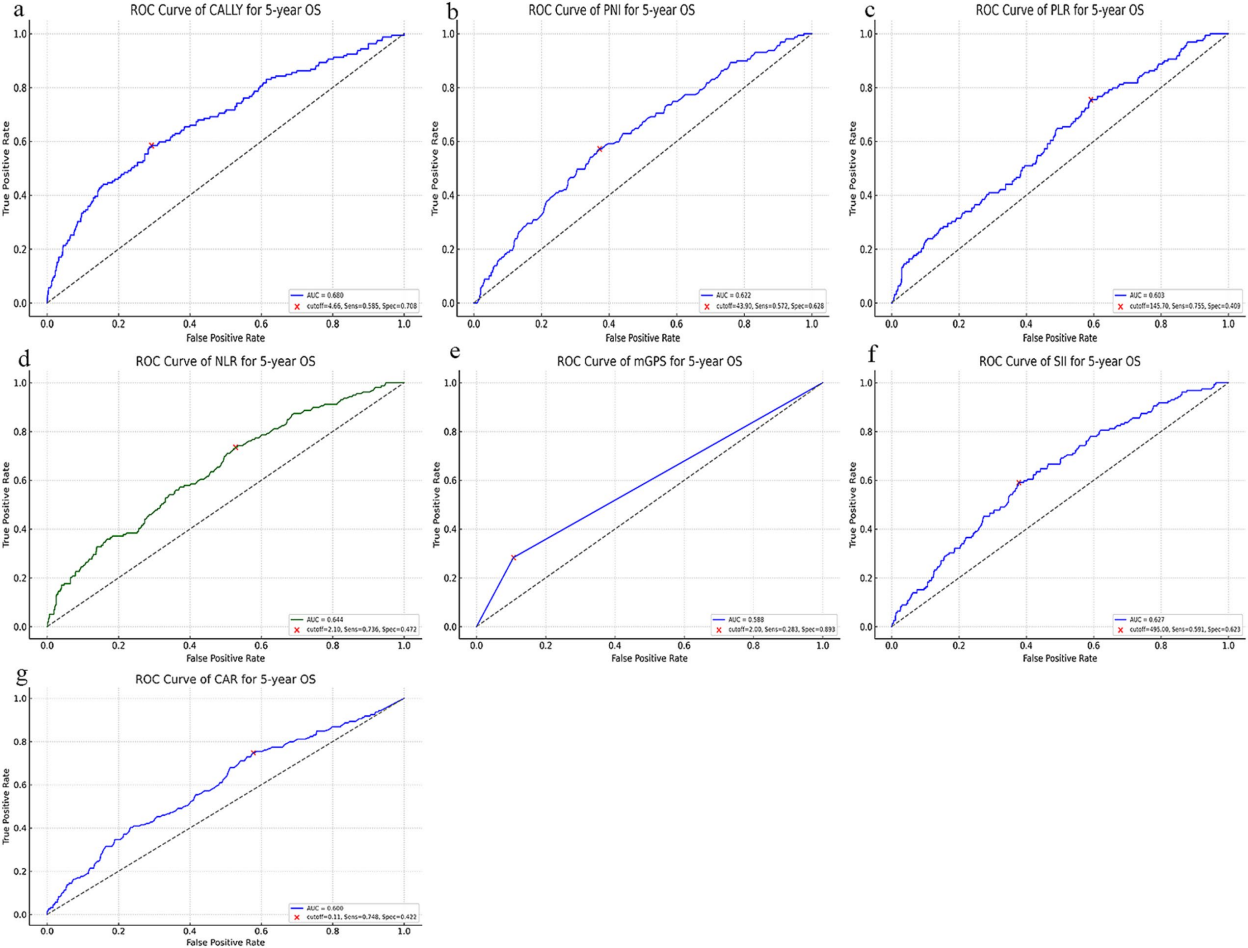
Receiver operating characteristic (ROC) curves of the CALLY index and related prognostic scores for predicting 5-year overall survival (OS) in patients with colorectal cancer. **(a)** ROC curve of the CALLY index. AUC: 0.680, Optimal cutoff: 4.66, Sensitivity: 0.585, Specificity: 0.708, 95% CI for AUC: (0.630, 0.730). **(b)** ROC curve of the prognostic nutritional index (PNI). AUC: 0.622, Optimal cutoff: 43.90, Sensitivity: 0.572, Specificity: 0.628, 95% CI for AUC: (0.574, 0.671). **(c)** ROC curve of the platelet-to-lymphocyte ratio (PLR). AUC: 0.603, Optimal cutoff: 145.70, Sensitivity: 0.755, Specificity: 0.409, 95% CI for AUC: (0.551, 0.654). **(d)** ROC curve of the neutrophil-to-lymphocyte ratio (NLR). AUC: 0.644, Optimal cutoff: 2.10, Sensitivity: 0.736, Specificity: 0.472, 95% CI for AUC: (0.598, 0.692). **(e)** ROC curve of the modified Glasgow prognostic score (mGPS). AUC: 0.588, Optimal cutoff: 2.00, Sensitivity: 0.283, Specificity: 0.893, 95% CI for AUC: (0.553, 0.626). **(f)** ROC curve of the systemic immune-inflammation index (SII). AUC: 0.627, Optimal cutoff: 495.00, Sensitivity: 0.591, Specificity: 0.623, 95% CI for AUC: (0.580, 0.672). **(g)** ROC curve of the C-reactive protein-to-albumin ratio (CAR). AUC: 0.600, Optimal cutoff: 0.11, Sensitivity: 0.748, Specificity: 0.422, 95% CI for AUC: (0.550, 0.646). Each panel displays the area under the curve (AUC), optimal cutoff value, sensitivity, and specificity.

ROC analysis confirmed superior performance of the CALLY index relative to mGPS, PNI, NLR, PLR, SII, and CAR. For 5-year OS, CALLY achieved the highest AUC, with similar results for DFS (Figure 3). Additionally, the C-index value of the CALLY index demonstrated stronger discriminatory ability compared to other indices (Supplementary Table 1). These findings underscore that CALLY more effectively integrates systemic inflammation, nutritional status, and immune competence than single-parameter indicators.

## 4 Discussion

In this comprehensive retrospective analysis of 957 patients with CRC who underwent curative resection, we examined the prognostic relevance of the preoperative CALLY index. Our findings demonstrate that the CALLY index is a reliable marker that can predict both short-term postoperative outcomes and long-term survival. Patients with reduced CALLY index not only exhibited a greater incidence of postoperative complications but also faced significantly worse OS and DFS. Importantly, the prognostic value of CALLY remained evident after multivariable adjustments that included TNM stage and histological subtype. CALLY demonstrated superior discriminative ability compared to established host-related indices, such as NLR and PLR, as validated by ROC analysis and C-index. These findings underscore the clinical significance of incorporating CALLY into prognostic evaluation, as it bridges tumor burden with systemic host status.

The biological plausibility of CALLY’s prognostic impact stems from its capacity to integrate three fundamental host domains: systemic inflammation, nutritional reserve, and immune competence. Each of the individual components contributes unique yet interconnected prognostic information. Elevated CRP levels are widely recognized as a surrogate marker of chronic inflammation, driven primarily by proinflammatory cytokines such as IL-6, IL-1β, and TNF-*α* (15). Persistent activation of the IL-6/STAT3 pathway, for instance, has been implicated in tumor-promoting processes including angiogenesis, epithelial-to-mesenchymal transition (EMT), invasion, and immune evasion (16). Hypoalbuminemia and lymphopenia are important markers of systemic nutritional and immune depletion. Hypoalbuminemia, another key parameter, not only reflects poor nutritional status but also mirrors systemic catabolism and chronic inflammatory consumption. Albumin depletion compromises collagen synthesis and tissue repair, thereby delaying wound healing, predisposing to infectious complications, and diminishing tolerance to chemotherapy or other systemic treatments (17). Hypoalbuminemia is typically associated with malnutrition, chronic inflammation, and impaired liver synthetic function, factors that influence the prognosis of CRC by altering immune system function (18). Specifically, low albumin levels are often correlated with elevated inflammation markers such as CRP, indicating the presence of systemic inflammation, which in turn affects immune responses and treatment tolerance. In CRC, chronic inflammation enhances immune evasion within the tumor microenvironment through proinflammatory cytokines, promoting the spread of cancer cells (19). Lymphopenia reflects a decline in immune function, particularly the impaired function of T cells and natural killer (NK) cells, which weakens tumor immune surveillance, leading to immune evasion and cancer progression (20). Therefore, hypoalbuminemia and lymphopenia are not only markers of nutritional and immune depletion but may also directly impact the prognosis of CRC patients by immunosuppression and promoting tumor progression. Together, these factors provide a compelling rationale for why CALLY, which simultaneously integrates these three axes, is a more reliable predictor than single-parameter indices.

The role of inflammation and immune status in cancer progression is well-recognized, with many studies evaluating the prognostic impact of various inflammation-related biomarkers in CRC. For example, the preoperative lymphocyte-to-monocyte ratio (LMR) has been shown to be a prognostic biomarker in recurrent CRC patients. Özkan et al. found that higher preoperative LMR was significantly associated with improved OS and recurrence-free survival (RFS) in recurrent CRC patients (21). This evidence supports the importance of systemic inflammation and immune status in CRC prognosis, further validating CALLY as a comprehensive biomarker that integrates these factors. Moreover, composite indices based on inflammation and nutrition have gained prognostic significance across various malignancies. A recent study by Yilmaz et al. demonstrated that the Naples Prognostic Score (NPS), which integrates inflammatory and nutritional markers, independently predicts OS and progression-free survival (PFS) in non-small cell lung cancer (NSCLC) patients (22). This study strengthens the contextual framework of the current manuscript, underscoring that the prognostic value of systemic inflammation and nutrition-based indices, such as CALLY, is supported across multiple tumor types.

Our results align with existing evidence regarding the prognostic utility of the CALLY index in gastrointestinal malignancies and further extend this evidence. Studies have shown that CALLY can predict worse survival outcomes and higher complication risks in patients with gastric cancer, hepatocellular carcinoma, and esophageal cancer, supporting its applicability as a prognostic biomarker across different cancer types (5, 8, 23).

Our findings are consistent with, and add to, the growing body of literature investigating CALLY in gastrointestinal malignancies. In CRC specifically, Yang et al. reported that the CALLY index was independently associated with OS and held a higher prognostic value than mGPS, NLR, SII, and PLR in a cohort of 1,260 CRC patients from the INSCOC database (12). In a more focused study on stage II–III CRC patients, Furukawa et al. found that a lower CALLY index (cutoff = 3.41) was associated with poorer OS (*p* = 0.008) and a trend toward worse RFS (*p* = 0.062) in those undergoing surgery (14). Takeda et al. similarly proposed that CALLY could be a prognostic biomarker for long-term outcomes in CRC patients undergoing surgery (13). More recently, another study demonstrating that higher CALLY index was significantly associated with improved OS and RFS, and multivariate Cox regression confirmed CALLY as an independent prognostic factor (24). Moreover, a meta-analysis by Wu et al., encompassing multiple digestive tract cancers including CRC, demonstrated that lower CALLY was strongly correlated with unfavorable survival outcomes (pooled HR for OS ≈ 1.97, *p* < 0.001) (11). Collectively, these consistent results across diverse study populations and methodological approaches reinforce the reliability of our findings and underscore the broad clinical applicability of CALLY as a prognostic biomarker in CRC.

The dual predictive capacity of CALLY-encompassing both surgical outcomes and survival endpoints-offers significant clinical benefits. By identifying high-risk patients preoperatively, CALLY can guide personalized interventions, including nutritional optimization and immunonutrition, reducing complications and improving recovery (25, 26). It can also refine patient stratification within ERAS protocols to ensure that limited resources are directed to those most likely to benefit (27).

From an oncological standpoint, CALLY complements TNM staging by providing additional insight into host biology (12, 13). While TNM staging remains indispensable for assessing tumor burden, it fails to account for the host’s systemic condition, which strongly influences treatment tolerance and prognosis (28). Incorporating CALLY into clinical decision-making could therefore refine adjuvant therapy strategies. Incorporating CALLY into clinical decision-making can improve adjuvant therapy strategies, particularly for low CALLY patients who may benefit from more aggressive regimens or closer surveillance.

The clinical significance of the CALLY index includes not only its prognostic value for OS and DFS, but also its broader application in patient management. In the perioperative setting, the CALLY index plays a critical role in risk stratification and personalized management. By integrating systemic inflammation, nutritional status, and immune competence, the CALLY index enables the identification of high-risk patients who may benefit from targeted preoperative interventions such as nutritional optimization, immune modulation, and enhanced monitoring (29). For low-CALLY patients, early interventions aimed at reducing inflammation and improving immune function can help reduce postoperative complications and enhance recovery. Moreover, the CALLY index provides valuable insights into a patient’s ability to tolerate surgery, which helps refine surgical decision-making and ultimately improve clinical outcomes. The ability to stratify patients based on CALLY index provides a powerful tool for perioperative management, ensuring that high-risk patients receive the necessary interventions, while avoiding unnecessary treatments for low-risk patients. This personalized approach to perioperative care not only improves surgical outcomes but also contributes to long-term survival and quality of life. Furthermore, the CALLY index is easily integrated into routine clinical practice, as it relies on simple, routinely collected laboratory data. This makes it a valuable tool for both developed and resource-limited settings, offering a cost-effective and accessible method for assessing patient prognosis and guiding perioperative management.

The implications of CALLY extend beyond surgery and adjuvant therapy. With the increasing adoption of immune checkpoint inhibitors in MSI-H/dMMR CRC, host immune competence is emerging as a key determinant of therapeutic efficacy (30, 31). In addition, the relationship between the CALLY index and key molecular markers in CRC, such as MSI status, KRAS/NRAS, and BRAF mutations, could provide deeper insight into the biological underpinnings of CRC and its response to treatment. These molecular markers are established prognostic and predictive factors, and their integration with CALLY could enhance predictive models. Because CALLY reflects both immune and inflammatory status, it could potentially serve as a predictive biomarker for immunotherapy responsiveness. This hypothesis merits exploration in prospective clinical trials of PD-1/PD-L1 inhibitors and combination immunotherapies. Furthermore, combining CALLY with molecular and genomic markers such as MSI, KRAS, and BRAF mutations, as well as circulating tumor DNA (ctDNA), could generate more comprehensive predictive models that align with the principles of precision oncology. Combining CALLY with molecular markers could pave the way for more tailored treatment strategies in CRC, potentially improving the precision of outcome predictions. Integrating CALLY with these molecular markers may allow for more personalized therapeutic approaches in CRC, improving the accuracy of predictions for patient outcomes. Integration with emerging “multi-omics” approaches-including transcriptomics, metabolomics, and immunoprofiling-may further enhance predictive accuracy and yield mechanistic insights into host-tumor interactions (32, 33).

An additional advantage of the CALLY index is its accessibility and affordability. Because it is derived entirely from routine laboratory measurements, it imposes no additional financial burden on patients or healthcare systems. This is particularly important in low- and middle-income countries, where access to costly molecular or genomic assays is limited (34). CALLY’s ease of calculation and universal availability make it an attractive option for widespread clinical implementation, facilitating global standardization of host-related prognostic assessment.

This study provides new insights into the prognosis of CRC by evaluating the CALLY index. Unlike previous studies, our research utilizes a large, single-center dataset comprising 957 patients, offering stronger statistical power and enhancing the generalizability of the findings. Furthermore, our study uniquely combines postoperative complications with long-term survival outcomes, providing a more comprehensive understanding of patient prognosis. In contrast, previous studies primarily focused on survival, while our research establishes a dual relationship between complications and survival. More importantly, our study includes more recent patient data, reflecting contemporary clinical practices and patient demographics, ensuring the timeliness and relevance of the findings. Therefore, we believe that this study significantly extends existing research by providing a more comprehensive and clinically practical method for CRC prognosis. Our study does not alter the basic evaluation function of the CALLY index but rather supplements and expands its specific application in CRC, offering a more precise risk assessment tool for future clinical practice and research.

Despite these strengths, several limitations warrant discussion. First, this study is a single-center retrospective analysis, with data derived from a cohort of patients at a single hospital, which may introduce retrospective bias. Selection bias, inherent in retrospective studies, may affect the representativeness of the patient population and confound the results, as it is not possible to fully control for all potential confounding factors. This may limit the generalizability of the findings. In addition, the assessment of postoperative complications and survival outcomes was not performed by clinicians blinded to the CALLY index groups. Although blinding was not employed in this study, we minimized potential ascertainment bias by relying on objective clinical data. Future studies should validate the CALLY index in multi-center prospective cohorts and consider incorporating blinding in both the assessment and analysis phases to further reduce the risk of bias. Second, although the optimal CALLY cutoff was determined using ROC analysis in this study, the cutoff was only validated in the same cohort of 957 patients, which introduces a risk of overfitting. Hence, further research should validate the cutoff in independent external cohorts to assess its reliability and generalizability. Additionally, prospective cohort studies can better control for confounding factors and further evaluate the prognostic value of the CALLY index through long-term follow-up. Third, the CALLY index was measured only once preoperatively, and its dynamic changes during the perioperative period or follow-up were not assessed. Dynamic monitoring of the CALLY index may provide further insights into host-tumor interactions and treatment responses. Therefore, future research should focus on the dynamic changes of the CALLY index and explore its prognostic value in treatment response and recurrence. Fourth, although we compared CALLY with several established inflammation- and nutrition-based indices, molecular and immunological markers were not included. Incorporating such biomarkers could enhance the comprehensiveness of prognostic models. Fifth, the inconsistency of the CALLY index cutoff across different studies constitutes a source of heterogeneity. The cutoff in our study was determined by ROC analysis and set at 4.66, showing good reliability when compared to the results of other studies. For instance, Yang et al. (12) set the CALLY cutoff at 3.41, while Takeda et al. (13) used 2.0 as the cutoff. Additionally, other studies have used different thresholds, such as 6.79 (29). These discrepancies highlight the need for standardization of the CALLY cutoff in future research, further validating the rationale behind the cutoff we selected. Sixth, laboratory variability across institutions in measuring CRP, albumin, and lymphocyte counts may also limit external comparability. Seventh, as our cohort was composed entirely of Chinese patients, further validation in ethnically and geographically diverse populations is necessary before global implementation. Finally, although we controlled for several known influencing factors, some potential confounders were not fully incorporated into the model in this study. For example, comorbidities (such as cardiovascular diseases and diabetes), smoking status, etc., may affect survival. These comorbidities and smoking status often influence the patient’s overall health status and treatment response, and failing to fully control for these factors may impact the relationship between the CALLY index and prognosis. Additionally, inflammation or nutritional interventions could also be important factors affecting survival. CRC patients often receive anti-inflammatory treatment or nutritional support, which can improve the patient’s health status and potentially impact prognosis. However, the current study did not fully consider the potential confounding effects of these factors on the CALLY index. Therefore, future studies should collect more detailed data on comorbidities, inflammatory responses, and nutritional interventions to further assess the impact of these factors on the prognostic ability of the CALLY index.

## 5 Conclusion

Our findings provide promising evidence that the preoperative CALLY index is a simple, cost-effective, and effective biomarker that independently predicts both postoperative complications and long-term survival in CRC. However, due to the single-center, retrospective design, the generalizability of these findings is limited, and further validation in multi-center, prospective cohorts is necessary. Compared with conventional inflammation- and nutrition-based scores, CALLY demonstrated superior prognostic accuracy. By capturing systemic inflammation, nutritional reserve, and immune competence within a single metric, CALLY offers a holistic perspective on host-tumor interactions that traditional staging systems cannot provide. Clinically, it holds potential as a preoperative risk stratification tool, an adjunct to TNM staging for tailoring adjuvant therapy, and a candidate biomarker for guiding immunotherapy. Its accessibility and affordability also make it particularly valuable in global oncology practice, including resource-limited settings. Future multicenter prospective studies should validate its prognostic role, standardize cutoff definitions, evaluate its predictive capacity for immunotherapy, ultimately advancing personalized CRC management and improving patient outcomes.

## Data Availability

The raw data supporting the conclusions of this article will be made available by the authors, without undue reservation.
