# Supplementary material for: CALLY index predicts survival and surgical outcomes in colorectal cancer
Source: Front Nutr. 2025 Dec 17;12:1723789. doi: 10.3389/fnut.2025.1723789 (PMC12753397; doi:10.3389/fnut.2025.1723789)
Supplement: Supplementary file 1 [file Table_1.docx]

Supplementary Table 1 C-index and 95% Confidence Interval for Prognostic Indices in Colorectal Cancer

| Variable | C-index | 95% CI Lower | 95% CI Upper |
| --- | --- | --- | --- |
| CALLY | 0.669 | 0.628 | 0.715 |
| PNI | 0.625 | 0.57 | 0.654 |
| PLR | 0.593 | 0.557 | 0.634 |
| NLR | 0.626 | 0.586 | 0.675 |
| mGPS | 0.587 | 0.541 | 0.627 |
| SII | 0.617 | 0.586 | 0.659 |
| CAR | 0.605 | 0.559 | 0.639 |

CALLY: C-reactive protein-albumin-lymphocyte; NLR: neutrophil-to-lymphocyte ratio; PLR: platelet-to-lymphocyte ratio; PNI: prognostic nutritional index; mGPS: modified Glasgow prognostic score; SSI: systemic immune-inflammation index; CAR: C-reactive protein/albumin ratio
